# Association of *ENAM* gene single nucleotide polymorphisms with dental caries in Polish children

**DOI:** 10.1007/s00784-016-1743-1

**Published:** 2016-02-24

**Authors:** Karolina Gerreth, Katarzyna Zaorska, Maciej Zabel, Maria Borysewicz-Lewicka, Michal Nowicki

**Affiliations:** Department of Paediatric Dentistry, Poznan University of Medical Sciences, 70 Bukowska Street, 60-812 Poznan, Poland; Department of Histology and Embryology, Poznan University of Medical Sciences, 6 Swiecickiego Street, 61-781 Poznan, Poland

**Keywords:** Dental caries, *ENAM* gene, primary dentition, single nucleotide polymorphism markers

## Abstract

**Objectives:**

The objective of this study was to prove the association between dental caries and single nucleotide polymorphisms (SNPs) in the *ENAM* gene.

**Material and methods:**

The research was carried out in 96 children (48 with caries and 48 counterparts free of this disease), aged 20–42 months, with 11–20 erupted teeth. All children were from four day nurseries located in Poznan. The study included the dental examination to select individuals to the research and oral swab collection for molecular evaluation. Seven selected SNPs markers of the *ENAM* gene were genotyped, five using TaqMan probe assay (rs2609428, rs7671281, rs36064169, rs3796704, and rs12640848) and two by Sanger sequencing (rs144929717 and rs139228330).

**Results:**

Statistically significant higher prevalence of the alternative G allele and the alternative GG homozygote in the control group in comparison with the caries group in SNP rs12640848 was observed, respectively, *p* = 0.0062 and 0.0010. Although the prevalence of the AG heterozygote was higher for the caries subjects in comparison with controls (OR = 2.9), and the result was statistically significant (*p* = 0.0010), the overall prevalence of the G allele for this SNP was significantly higher in control group (OR = 2.3; *p* = 0.0062).

**Conclusions:**

The study revealed the strong association between rs12640848 marker of *ENAM* gene and caries susceptibility in primary teeth in children from Poznan.

**Clinical relevance:**

The presence of SNPs in the ENAM gene may be important as suspected predictive factor of dental caries occurrence in children.

## Introduction

Dental enamel is of epithelial origin and the most highly mineralized tissue. It is different than other vertebrate hard tissues, such as dentine or bone, because of non-collagenous nature and inability of remodeling and resorption [1]. In spite of the hardness, enamel is damaged quite quickly by dental caries that is infectious disease [2]. Once it is affected by carious process, the enamel may not regenerate.

The enamel-specific matrix proteins such as amelogenin, enamelin, and ameloblastin are secreted by secretory stage ameloblasts and are critical for proper enamel mineralization [3]. After reaching the final dimension of the enamel, those cells undergo transformation to maturation stage ameloblasts and degradation as well as reabsorption of matrix proteins begins. Such events lead to enamel hardening due to enamel crystals increasing.

There is strong argument for genetic contribution in caries. Upon genome-wide scan and linkage analyses for dental decay, several gene candidates have been proposed to be associated with caries susceptibility [4]. Among them, genes being in charge of formation of the tooth enamel have been suggested to be responsible for the disease. Literature data revealed the association between genetic variations in different matrix proteins of enamel and the process [5–9].

The aim of this paper is to reveal if there is an association between variations in enamel-specific protein such as enamelin (SNPs within the *ENAM* gene) and caries occurrence in children from Polish population.

Thus, we investigated seven SNPs (markers) within *ENAM* gene (rs144929717, rs139228330, rs2609428, rs7671281, rs36064169, rs3796704, and rs12640848) that were investigated previously by other researchers [5–9]. However, none of the authors have taken into consideration small children with milk dentition under the age of 3 years as a total examined population. The youngest studied population of children, aged 3–5 years, was described by Patir et al. [6]. Furthermore, the patients examined earlier by other researchers were of different age, sometimes with different type of dentition, from different environments.

## Material and methods

### Study group

The research was carried out in children from four day nurseries that constitute one institution, located in the city of Poznan (Wielkopolska Province, central-west Poland) and included dental examination and molecular analyses of the biological material. Before the study, it was assessed that 321 children attended aforementioned establishment. Information concerning the research was provided to the staff of the day nurseries and each parent/caregiver received written information concerning the purpose of the research with consent form. After a few days, 265 parents’ consents were collected. The researchers visited each day nursery 2–3 times to carry out check-ups and biological material collection, since some of the children were absent during the day of examination. Finally, 262 children had dental examination, because three individuals were absent during each visit. In 48 subjects, dental caries were diagnosed and they were classified as study group (“cases”). From the other 214 subjects, the control group of 48 children was selected. In general, individuals in the study and control groups were matched by gender, age, and the number of erupted teeth.

From the individuals of the study and control groups, the oral swab was collected. In some cases when the quality of the biological material for molecular analysis was poor, the sample was taken 2–3 times. The specimens from children of other than Caucasian ethnic group were excluded from this study.

Finally, the study group included 48 children with caries (25 females—52.08 % and 23 males—47.92 %), aged from 20 to 42 months (the mean age and standard deviation were 30.58 ± 5.91). The participants had from 11 to 20 erupted teeth (18.56 ± 2.29). The control group composed of 48 counterparts (24 females—50.00 % and 24 males—50.00 %) that were free of carious disease, aged from 20 to 42 months (29.85 ± 5.48), with 12–20 erupted teeth (18.14 ± 2,16).

### Clinical examination

Oral examination was carried out, from April to June 2014, in the day nursery, in those children whose parents gave their written consent for dental check-up as well as oral swab collection. The participation of each child in the study was voluntary. Clinical examination was not performed, if the child refused to participate or failed to cooperate. The children were examined without any pharmacological preparation. Teeth were evaluated by one trained and calibrated dentist, specialist in pediatric dentistry, in the classroom, in an artificial light, with the use of a dental mirror and a probe, after calibration by an experienced specialist. Radiograph of the dentition was not taken. Dental evaluation concerned the occurrence of carious cavities and initial (incipient) caries lesions (non-cavitated, white spot). The intra-examiner agreement was evaluated by second dental examination in a group of 10 children after 2 weeks, with a κ of 1.00.

### Samples collection

Biological material was obtained from 96 study subjects during dental examination. Samples were collected from buccal swabs, which were provided to each subject in sterile packs. The inside of the mouth was rubbed at least ten times from each side of both cheeks and then the swab was placed inside the 1.5-ml Eppendorf tube. Then, the plastic stick was cut off and the Eppendorf tube was placed at +4 °C in a portable fridge until DNA extraction. Genomic DNA was extracted from the swabs the same day as it was collected, using EXTRACTME DNA Swab & Semen Kit from Blirt S.A. Samples were first centrifugated at short spin, and the purification of DNA was carried out according to the manufacturer’s instruction. The DNA samples were stored in −20 °C until further analyses.

### Molecular analysis

We performed genotyping of seven known to date from the literature single nucleotide polymorphisms (SNPs) in *ENAM* gene, with putative role in caries susceptibility according to the previous research, in 96 subjects, in total. Three of those variants were placed in intron regions (rs144929717, rs139228330, and rs12640848 in intron 4, 7, and 8, respectively) and did not change the amino acid sequence. All other four SNPs were placed in exon 9. One of them (rs36064169) was synonymous, while the other three variants (rs2609428, rs7671281, and rs3796704) were missense polymorphisms and changed the amino acid sequence of the protein.

Genotyping of polymorphisms 3–7 was assessed by the TaqMan MGB probes assay (TaqMan SNP Genotyping Assays, Life Technologies). The TaqMan probes were labeled at the 5′ end with the FAM and VIC reporter dyes, according to the wild and alternative allele of a given SNP and included a non-fluorescent quencher at the 3′ end. The real-time PCR was performed following the manufacturer’s protocol on the 96-well optical reaction plate, using 12.5 μl of TaqMan Genotyping Master Mix, 1.25 μl of 20× probe, and 11.25 μl of genomic DNA, in a 25-μl total reaction volume. DNA samples were adjusted to 10 ng per 11.25 μl by diluting the sample with appropriate amount of nuclease-free water (Ambion), while the probes at concentration 40× were diluted to a 20× working stock with 1× TE buffer. The cycling conditions were an initial denaturation at 95 °C for 10 min followed by 40 cycles of denaturation at 92 °C for 15 s and annealing/extension at 60 °C for 1 min, and the assay was carried out in an 7900HT real-time PCR System (Applied Biosystems). TaqMan Allelic Discrimination data were captured using the SDS software (Applied Biosystems). The results were shown as the increase in the fluorescent signal from only one of two dyes (FAM or VIC, separately) or from both dyes at the same time. It indicated the presence of one allele (homozygote) or two alleles (heterozygote).

Due to the lack of probes for SNPs 6 and 7 in the offer of Life Technologies at the time of analyses, we performed Sanger sequencing of short regions containing those two variants. PCR amplification was performed at Veriti 96-well Thermal Cycler (Applied Biosystems) using Fast Start Taq DNA Polymerase Kit (Roche) and specific primers designed using Primer3 free online software. The reactions were carried out in a total volume of 12.5 μl containing 10× Taq DNA Polymerase buffer with MgCl2, 5× GC-rich solution, 0.24 mM dNTPs, 0.5 μM of each primer, 1 unit of Taq Polymerase, and 20–40 ng of genomic DNA. The cycle conditions were an initial denaturation at 94 °C for 4 min, followed by 35 cycles of denaturation at 95 °C for 30 s, annealing at 59 °C for 1 min, and extension at 72 °C for 30 s, with final extension at 72 °C for 7 min. PCR primer sequences are available on request.

PCR products were purified using membrane Millipore plates and used as templates in PCR-sequencing reamplification. It was performed at Veriti 96-well Thermal Cycler, using BigDye Terminator v3.1 Cycle Sequencing Kit (Life Technologies) and one of the specific primers (Reverse for SNP 6 and Forward for SNP 7). Reamplification products were purified with EDTA and ethanol precipitation and separated by capillary electrophoresis using ABI 3130 sequencer (Applied Biosystems). Sequencing Analysis 5.2.0 software was used to analyze the sequences, using the reference sequence of *ENAM* gene from Ensembl database.

### Statistical analysis

Genotypes obtained in the study, both from TaqMan probe assay and Sanger sequencing, were calculated and the chi-square test and deviation from Hardy-Weinberg equilibrium (HWE) were performed. Genotype and allele frequencies for each SNP were evaluated and compared between study and control groups using the Fisher’s exact test. Odds ratio values (OR) were also evaluated and *p* < 0,05 was considered statistically significant. Additionally, we used Haploview 3.2 software to obtain *ENAM* gene structure. Linkage disequilibrium values (LD) were calculated as *R*^2^ value and Gabriel et al. algorithm was used [10].

## Results and discussion

We have chosen enamelin in our study since it is the largest protein of enamel with expression highly limited to developing tooth [1].

As far as we know from the literature, none of the previous researchers examined all seven SNPs in the *ENAM* gene showed in the present study in one such a homogenous population of children under the age of 3 years with primary dentition that attend to one institution. It must be emphasized that the examined (case and control) population was properly matched, with the same age and proportion of females and males. The selected group was also homogenous with no ethnic, cultural, demographic, and regional differences since all children lived in one city (Poznan). Examined individuals attended one group of day nurseries, so one may expect that the children have similar environmental factors such as dietary and oral hygienic habits since some of them spend all day long at this institution. The children had similar access to oral health care. Moreover, the water in Poznan is not additionally and artificially fluoridated. Needless to say that oral examination with molecular analysis was carried out in a short time of 3 months.

Upon the genotype calculation, two SNPs were not differentiated and comprised only wild homozygotes in both caries and control groups (100 % of the CC homozygotes in rs2609428 and 100 % of the CC homozygotes in rs36064169) and were excluded from further statistical analyses. The other five SNPs were differentiated and showed the presence of wild-type homozygotes as well as alternative homozygotes and/or heterozygotes.

Interestingly, we observed statistically significant higher prevalence of the alternative G allele and the alternative GG homozygote in the control group in comparison with the caries group in SNP rs12640848 (results shown in Table 1). The prevalence of the GG genotype was ninefold higher in control subjects than in caries patients, which strongly indicated for the protective variant against caries incidence in children of our study group for this genotype. Although the prevalence of the AG heterozygote was higher for the caries subjects in comparison with controls (OR = 2.9), and the result was statistically significant (*p* = 0.0010), the overall prevalence of the G allele for this SNP was significantly higher in control group (OR = 2.3; *p* = 0.0062). This could firmly indicate that the G allele and the GG genotype polymorphic variants in SNP rs12640848 might be a protective variant against caries incidence in children of studied population. In our opinion, the results indicated this polymorphism to be a protective variant against caries incidence in children of studied population. Moreover, as far as we are concerned, this is the first study which investigates genetic susceptibility for caries development in such a homogenous group of small children under the age of 3 years from Polish population.

**Table 1:** Frequencies and odds ratio values for chosen SNP in *ENAM* gene

Variation			Caries patients	Controls		*p* value	OR (95 % CI)
rs12640848	Genotype frequency		(*n* = 48)	(*n* = 48)			
	AA		8 (0.17)	4 (0.08)	*Cr* vs. *Ctrl*	0.2253	2.2 (0.6–7.9)
	AG		37 (0.77)	26 (0.54)	*Cr* vs. *Ctrl*	0.0199*	2.9 (1.2–6.9)
	GG		3 (0.06)	18 (0.38)	*Ctrl* vs. *Cr*	0.0010*	9 (2.4–33.2)
	Allele frequency						
	A		53 (0.55)	34 (0.35)			
	G		43 (0.4)	62 (0.65)	*Ctrl* vs. *Cr*	0.0062*	2.3 (1.3–4)

* p<0.05

**Fig. 1:**
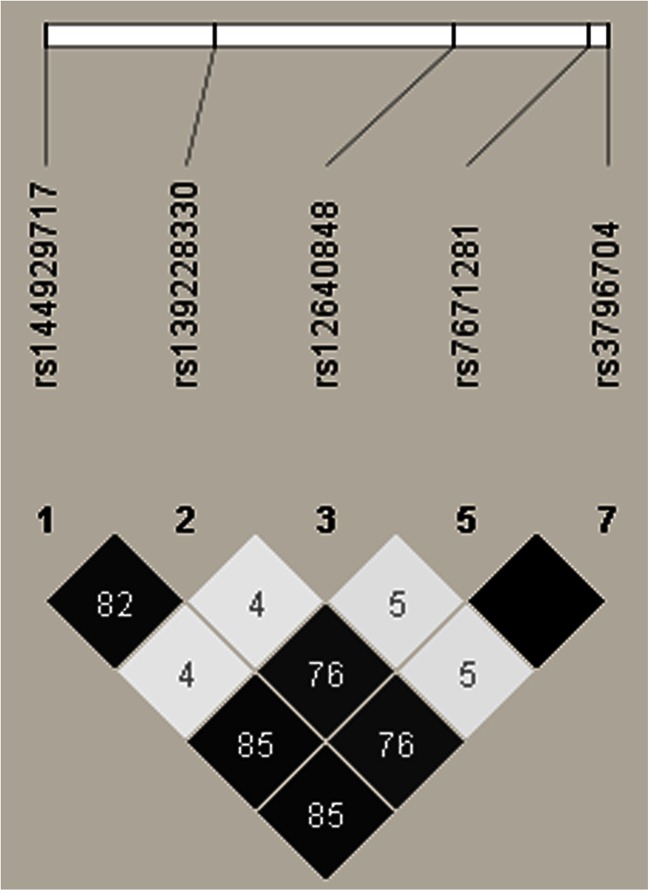
The 100% LD between SNPs 5 and 7

The results are in agreement with the data of Shimizu et al. [5]. The authors carried out the research in five populations, living in Philippines, Turkey, Argentina, and two in Brazil, and detected association also between rs12640848 in *ENAM* and caries experience in the Brazilian cohort of children, between 10 and 14 years of age, from Curtiba. On the other hand, Wang et al. did not found any correlation between primary tooth caries and SNPs (rs12640848, rs3796704, and rs7671281) in *ENAM* gene in a sample of examined children from parent-offspring trios (two parents and one child), aged 4–7 years, within the Iowa Fluoride Study [8]. It is intriguing that Chaussain et al. showed high association between rs12640848 and caries susceptibility (OR = 3.89; 95 % CI = 1.47–10.33) but after adjustment of environmental agent, the OR was found to be non-significant [7]. In the same study, correlation between rs3796704 in coding region of *ENAM* and caries was found. Interestingly, this variation caused amino acid change from arginine to glutamine and according to the authors such substitution is not frequently found and has never been observed during mammalian evolution. However, our study did not show any statistically important differences in genotype or allele frequencies between study and control subjects. Moreover, Chaussain et al. showed that one haplotype which consisted of six SNPs (rs144929717, rs139228330, rs2609428, rs7671281, rs36064169, and rs3796704)—all examined in our study as well, strongly corresponded to caries susceptibility, especially concerning the C allele in rs7671281 and the A allele in rs3796704. Although our results proved indeed strong correlation between those two markers (100 % LD shown in Fig. 1), none of those two variants separately corresponded to caries susceptibility. However, it is difficult to compare our data with those from other studies because the individuals are of different age and ethnicity as well as with different dentition.

We did not observe differences in allele and genotype frequencies for the other polymorphisms studied. The odds ratio values were oscillating around the neutral “1” value, and the results were not statistically significant (data not shown). The nucleotide substitutions in three of those SNPs (rs2609428, rs7671281, and rs3796704) changed amino acid sequence of a protein. It is worth mentioning that one polymorphic variant rs7671281 caused amino acid substitution from polar hydrophilic threonine to non-polar hydrophobic isoleucine, which not only could influence folding of messenger RNA (mRNA) molecule but also was likely to change the structure and function of ENAM protein. However, there were no differences in allele and genotype frequencies between the two study groups and we observed nucleotide substitution (C > T) both in caries patients and in control subjects, so presumably the amino acid change was not related to different protein behavior and interaction in patients with caries as expected.

There were also no differences in genotype and allele frequencies between male and female subjects both in the study and control groups (data not shown).

Additionally, the case-control association study test performed using Haploview showed strong linkage disequilibrium values (LD) in the *ENAM* gene. There was 100 % LD between SNPs 5 and 7 as well as strong statistically significant linkage between SNPs 1, 2, 5, and 7 (Fig. 1). There were higher LD values for the pairs of SNPs 1–5 and 1–7 solely in the control group vs. the patients with caries (100 vs. 61 % for both pairs) as well as slightly higher LD value for the pair 1–2 in the group with caries in comparison with the controls (100 vs. 74 %), which could indicate a bit different pattern of genotype distribution in control and study subjects (data not shown). The Haploview analysis did not reveal the presence of any haplotype block, nevertheless it showed quite strong pairwise linkage between most of studied polymorphisms in *ENAM* gene.

Changes in the genes that encode proteins of the tooth enamel generally cause malformation of this tissue without affecting other parts of the organism [2]. Disturbances during the secretory stage of enamel formation result in such changes as hypoplastic or pathologically thin enamel [2]. Thus, one may suspect that such defects within enamel could predispose this tissue to caries since it is the outer layer of the tooth where the carious process begins first. It was also corroborate in the literature. As Patir et al. suggested, the enamel in individuals with genetic variations in genes involved in their formation may have higher levels of mineral loss under acidic condition as well as biofilm deposition and bacterial attachment is facilitated in such a situation [6]. Moreover, Chaussain et al. analyzing the data of their research and other studies and suggested that particularly the variant rs3796704 may cause some changes within the microstructure of enamel that facilitate the progression of the caries through this tooth tissue [7]. However, in the present study, we did not notice rs3796704 marker as having an influence on caries development between study and control groups.

It is worth mentioning that there is one publication in the literature concerning an aspect of dental caries and polymorphism in ENAM gene in the Polish population [9]. Olszowski et al. studied the possible associations between marker ENAM C2452T (i.e., rs36064169) and carious disease in children, aged 5 and 13 years, from Szczecin (northwestern Poland) [9]. The authors carried out the research with the division of each age group into two subgroups: with higher and lower caries experience. However, the investigation revealed no association between ENAM C2452T mutation and caries since there were only two heterozygotes in 13-year-old children with higher caries experience and not any in other subgroups. This is in agreement with the findings of the present study but we found no polymorphisms within this marker in the total examined population.

In conclusion, the present study demonstrates rs12640848 variant in *ENAM* gene is a strong candidate gene for caries susceptibility in primary teeth of children from Poznan. One may suggest these SNPs as a potential predictor factor for dental caries diagnosis in deciduous dentition. However, further replication of the present analysis with the use of larger sample size should be carried out to improve this theory. On the other hand, comparison with small children from other Polish cities as well as foreign population, including different races, would be valuable.
